# Cell-in-Cell Events in Oral Squamous Cell Carcinoma

**DOI:** 10.3389/fonc.2022.931092

**Published:** 2022-06-30

**Authors:** Leonardo de Oliveira Siquara da Rocha, Bruno Solano de Freitas Souza, Daniel W. Lambert, Clarissa de Araújo Gurgel Rocha

**Affiliations:** ^1^ Gonçalo Moniz Institute, Oswaldo Cruz Foundation (FIOCRUZ), Salvador, Brazil; ^2^ Department of Pathology and Legal Medicine, School of Medicine, Federal University of Bahia (UFBA), Salvador, Brazil; ^3^ Center for Biotechnology and Cell Therapy, D'Or Institute for Research and Education (IDOR), Salvador, Brazil; ^4^ School of Clinical Dentistry, The University of Sheffield, Sheffield, United Kingdom; ^5^ Department of Clinical Propedeutics, School of Dentistry, Federal University of Bahia (UFBA), Salvador, Brazil

**Keywords:** cell-in-cell formation, cell cannibalism, entosis, emperipolesis, oral squamous cell carcinoma

## Abstract

For over a century, cells within other cells have been detected by pathologists as common histopathological findings in tumors, being generally identified as “cell-in-cell” structures. Despite their characteristic morphology, these structures can originate from various processes, such as cannibalism, entosis and emperipolesis. However, only in the last few decades has more attention been given to these events due to their importance in tumor development. In cancers such as oral squamous cell carcinoma, cell-in-cell events have been linked to aggressiveness, metastasis, and therapeutic resistance. This review aims to summarize relevant information about the occurrence of various cell-in-cell phenomena in the context of oral squamous cell carcinoma, addressing their causes and consequences in cancer. The lack of a standard terminology in diagnosing these events makes it difficult to classify the existing cases and to map the behavior and impacts of these structures. Despite being frequently reported in oral squamous cell carcinoma and other cancers, their impacts on carcinogenesis aren’t fully understood. Cell-in-cell formation is seen as a survival mechanism in the face of a lack of nutritional availability, an acid microenvironment and potential harm from immune cell defense. In this deadly form of competition, cells that engulf other cells establish themselves as winners, taking over as the predominant and more malignant cell population. Understanding the link between these structures and more aggressive behavior in oral squamous cell carcinoma is of paramount importance for their incorporation as part of a therapeutic strategy.

## 1 Introduction

Cell-in-cell (CIC) structures are commonly defined as morphological findings that result from one or more cells being inside another. Despite these events being considered a frequent finding, there isn’t a consensus on the origin and/or the consequences of these events. Different cell engulfment processes have been described, such as cannibalism, entosis and emperipolesis; these appear to differ in aspects such as formation mechanism, cell-cell relationship, and inner-cell fate. However, due to the lack of well-established definitions for each of these terms, many authors disagree as to what defines each CIC event (1, 2), which leads to multiple classifications and confused nomenclature of CIC, and of the mechanisms behind their formation.

CIC formation in cancer can have important effects in tumor progression. Among the several cancers in which these structures have been found, oral squamous cell carcinoma (OSCC) is an invasive and aggressive cancer which represents the most common type of oral cancer worldwide (3). Its mortality rate five years after diagnosis is of almost 30% (4). The aggressiveness of this tumor is the main cause of its high mortality and morbidity, associated with a lack of effective chemotherapeutic options available. Despite advances in research, the complex biology behind this tumor is still not fully understood, and an insight into interactions between cells can aid in the comprehension of the way OSCC progresses. In this context, understanding of CIC formation and its effects in OSCC is relevant in establishing correct prognosis markers and identifying possible therapeutic targets. To our knowledge, among several reviews describing the general understanding of CIC structures in the last five years, none of them focus on the relation between these structures and OSCC. The aim of this review is to summarize the current understanding of CIC structures, their different possible occurrences, and their impacts on tumor progression, particularly in the context of OSCC.

## 2 Cell-in-Cell Structures

**Figure 1:**
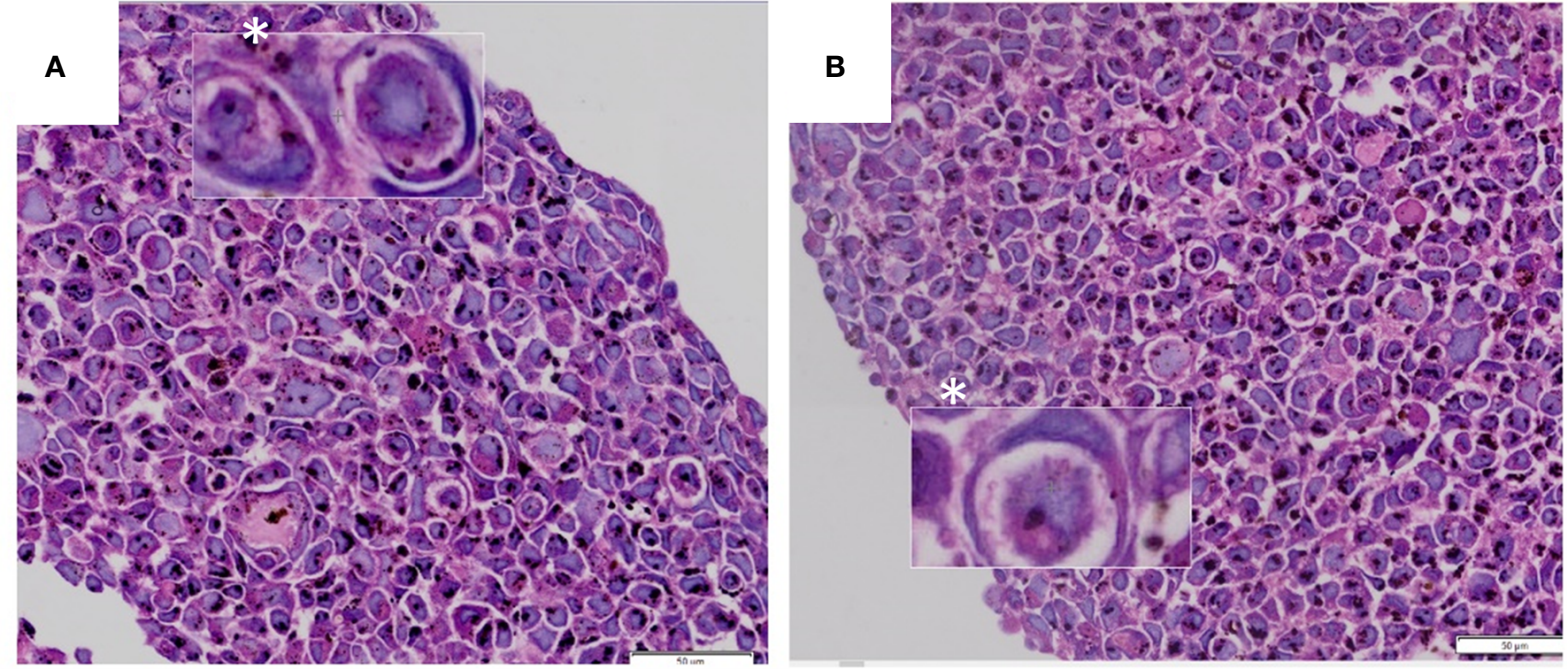
**(A**, **B)** CIC structures exhibiting morphological appearance of “signet ring” or “bird’s eye” cells. Images from tumor n3D spheroid cultures from the authors’ archive.

A CIC structure is morphologically identified as the presence of one or more living cells encapsulated within another, residing inside a vacuole that pushes the outer cell’s spindle-shaped nucleus towards its periphery (5, 6) (**Figure 1**). This feature was first reported over a century ago (7–9), when little was known about its composition, mechanism of biogenesis or the implications of its existence. Subsequently, studies have shown that CIC events are easily identifiable through hematoxylin-eosin staining and are common events in malignancies (10, 11), such as lung cancer (12), breast cancer (13, 14), melanoma (15, 16), and adenocarcinoma (17, 18), also having been detected in benign tumors (19).

Historically, terms such as “bird’s eye cells’’ (20) and even more commonly “signet-ring cells’’ (15, 21, 22) were used to describe similar morphological findings, in which intracellular vacuoles and/or cytoplasmic inclusions displaced the nucleus and changed its shape. As more cases of “signet-ring cells’’ were reported, authors began questioning the specificity of this term, suggesting that this morphological finding could represent different entities, such as mucin-producing cells (23–25), further emphasizing the importance of adequate methods to perform differential diagnosis. CIC identification in several tumor types became more consistent with time, through advances in light microscopy, electron microscopy and photograph-activation localization microscopy (26–30).

Despite this, due to a lack of precedents, different CIC findings were initially named and explained according to each author’s own understanding. At first, all cell engulfment events were recognized as cell cannibalism. As more cases were found, specificities began to be identified: some cannibalism events were only among identical cell types (homogenous cannibalism), while other events involved different cell types (heterogenous cannibalism). Once investigation began on the mechanisms behind CIC events, it was discovered that not all cases were the same. In a non-consensual manner, new terms and classifications appeared: what some authors described as cannibalism, others understood as entosis; what some called entosis, was explained by others as emperipolesis; and so forth. The issue with the classification and description of the physiopathology behind CIC structures persists, hampering their characterization and identification.

### 2.1 Cell-in-Cell Formation Mechanisms

**Figure 2:**
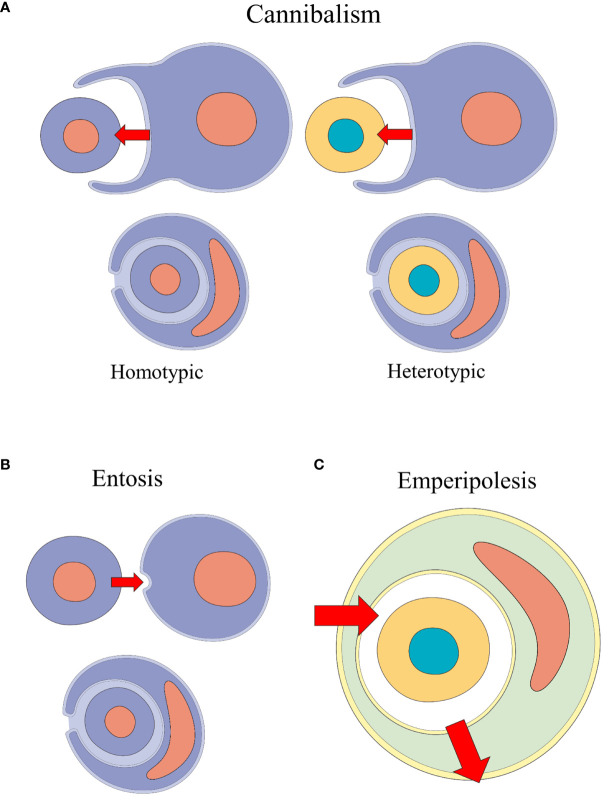
Schematic representation of the CIC formation mechanisms performed by non-professional phagocytic cells: cannibalism, entosis and emperipolesis. **(A)** Homotypic and heterotypic cannibalism events: the outer cell engulfs the inner cell. **(B)** Entosis event: one cell of the same phenotype invades another. **(C)** Emperipolesis: heterotypic cells transit inside other cells.

Many CIC events were identified according to their etiology and to target cell-specific processes, using different terms - e.g., tumor-cell phagocytosis (12, 31, 32) and erythrophagocytosis (33–36). With the rise of terms such as cannibalism, emperipolesis and entosis, many authors struggled to precisely determine which processes were involved in CIC events (37). Classification and definition behind CIC formation events remain unclear (2). **Table 1** summarizes the plurality behind CIC processes and their descriptions and **Figure 2** illustrates the main types of formation mechanisms behind these structures.

**Table 1:** Different characterizations given by authors to cell-in-cell formation processes.

Term	Description	Supporting authors
**Cannibalism**	Active engulfment of a cell by another, generally leading to the internalized cell’s destruction.	Brouwer et al. (38); Lugini et al. (39); Krajcovic and Overholtzer (40); Bartosh et al. (14); Gottwald et al. (18).
	A cell engulfed within another cell with a crescent-shaped nucleus.	Fais (30); Sharma and Dey (41); Barresi et al. (42); Jain et al. (43); Wang et al. (44); Siddiqui et al. (45).
	May occur in a homotypic and heterotypic manner.	Cano et al. (17); Gupta et al. (1); Suwasini et al. (46)
	Not exclusive to malignant tumors.	Fernandez-Flores (19); Sarode and Sarode (47).
**Entosis**	Active cell invasion mechanism resulting in non-apoptotic lysosome-mediated death of the inner cell	White (48); Sharma and Dey (41); Cano et al. (17); Barresi et al. (42); Siddiqui et al. (45); Wang et al. (49).
	One of the possible more specific mechanisms behind cell cannibalism	Krajcovic and Overholtzer (40); Sun et al. (50); Schmid et al. (16); Tonnessen-Murray et al. (39); Almangush et al. (51).
	Exclusively homotypical	Cano et al. (17); Jain (43); Gupta et al. (1).
	Not exclusive to malignant tumors.	Sarode and Sarode (52); Fais and Overholtzer (53).
**Emperipolesis**	General movement of cells passing through and within each other.	Humble et al. (54); Chemnitz and Bichel (27); Sarode et al. (55); Siddiqui et al. (45).
	Brings no damage or consequence to involved cells.	Brouwer et al. (38); Barresi et al. (42).
	Exclusively heterotypical, and the inner cell is hematopoietic.	Sharma and Dey (41); Jain (43); Sarode et al. (55); Gupta et al. (1).
	Overall definition for all cell-in-cell events formed by cannibalism and entosis.	Overholtzer and Brugge (5).

Recently, Borensztejn et al. (6) proposed a classification system for CIC structures based on their initiation mechanism. CIC structures are either formed through endocytosis-like steps, as is the case with cell cannibalism, phagoptosis and enclysis); or through inner-cell invasion, in entosis and emperipolesis. Further classification is based on molecular mechanisms and the phenotypical relation between the cells involved.

#### 2.1.1 Cannibalism

The term “cell cannibalism” was first used by von Leyden to collectively describe homotypic CIC structures (56). It is frequently described as the ability of a cell to engulf another cell or as the morphological finding of one cell or more contained within another (30, 41, 42). Morphologically, it presents itself as a cell with a crescent-shaped nucleus that has been pressed against the outer-cell membrane by the vacuole containing the inner-cell (30, 44, 57). This definition is similar if not equal to the one used to describe all CIC structures, and the generic and inconsistent use of this term generates confusion in diagnosis, as it erroneously classifies all CIC structures as cannibalism events. In fact, some authors use the term cannibalism in a broad sense to describe the overall idea of a cell engulfment event (40, 58), for example in benign giant cell tumors (19).

Cell cannibalism can be limited to describe CIC structures which form by active engulfment of a cell by another cell, resulting in the inner cell’s destruction (38, 39, 56). Cell cannibalism is considered by many authors a feature of malignant cells (30, 59, 60). Furthermore, several authors characterize cannibalism as a feature typical of metastatic as opposed to primary cancers (59). Cannibalism among the same type of cells is defined as homotypic cannibalism (17, 44) and seems to be related with elimination of tumor cells, being further associated with a better prognosis in pancreatic carcinomas (17). Heterotypical cannibalism, or xeno-cannibalism, on the other hand, occurs among cells with different phenotypes and is described as a more aggressive behavior performed by cancer cells both *in vivo* and *in vitro* (45, 59). Cannibalized cells are engulfed alive, and may be found intact within the outer cell, or undergoing degradation (30, 52).

The cannibalism process was initially described by Brouwer in 1984 (38). The “cannibal” cell will attach itself to a surrounding cell, engulfing it within its cytoplasm. The inner cell soon becomes surrounded by a membrane originating from the outer cell (61). As this occurs, its nucleus becomes compressed, semilunar, and pushed towards the periphery of the cell, while the engulfed cell maintains its conventional shape. Once the process is finished, the inner cell eventually dies by apoptosis, which is suggested by the lack of Bcl-2 (52) and presence of apoptosis markers (e.g., caspase-3) (42).

The completion of these steps depends on specific proteins and pathways. Lugini et al. (59) suggest that ezrin, an action-binding protein from the ezrin-radixin-moesin family (62), might be the connection between the actin cytoskeleton, responsible for the morphological execution of cell engulfment, and the caveolin-1-enriched endolysosomal vesicles found in cannibal cells. A dynamic and articulated structure between caveolin-1, ezrin and actin lead to the formation of caveolae-enriched endosomes, known as caveosomes. In addition, the pH of these intracytoplasmic vesicles was found to be lower in cannibal cells, which also expressed high levels of ATPases and proteolytic enzymes (cathepsin B), which suggests the caveosomes as protagonists behind the resulting digestion in the cannibalism process (43, 59).

High expression of TM9SF4, a member of the protein family TM9, is considered a differential marker for cannibal cells (63). This molecule is known for its role in pH regulation in intracellular vesicles (64) and for its involvement in cannibalism behavior of unicellular organisms (65). In this context, the maintenance of an acid environment within the caveosome is crucial for digestion of the inner cell, corroborating to the importance of these proteins in the process.

Cannibal cell activity is a part of a complex mechanism intimately related to the tumor microenvironment (TME) that can influence tumor progression. Stromal cells and surrounding non-tumoral cells appear able to secrete mediators that induce CIC formation (53). CIC structures are more frequent upon presence of inflammatory mediators such as IL-8 (66) and IL-6 (44). In this manner, cell cannibalism has been described as a survival mechanism in adverse TME conditions, such as low nutrient supply, hypoxia, and acidity (30, 41, 43, 47, 67).

Several authors have identified an increase of heterotypic cannibalism in serum-free cultures (46, 59, 68). This suggests that, in the face of starvation, tumor cells might turn to eating their non-tumoral counterparts to stay alive (47, 59, 62). Cannibal behavior in non-professional phagocytic cells seems to be triggered non-specifically by touch. Malignant tumor cells can “absorb” and feed on any neighboring bodies - whether alive or dead - upon nonspecific contact with their external membrane, in a maneuver described as a “quicksand-like” movement (17, 59).

The acidic tumor microenvironment also plays a pivotal part in influencing cannibalism activation. Metabolic changes, such as the Warburg effect, result in an acid microenvironment, which promotes further development of acid-resistant cell populations (61). Lugini et al. (59) reported a higher survival of metastatic cannibal cells in low pH cell culture condition, suggesting that acidity acts as a mechanism of selection for cannibal cancer cell clones.

Tumor cell cannibalism has also been suggested as an immune evasion mechanism (16, 59). By engulfing and digesting neutrophils, lymphocytes and erythrocytes, tumor cells inactivate their victims, dodging cell-dependent immune defense mechanisms (2, 62). Besides, ezrin has previously been associated with tumoral engulfment of lymphocytes (69), as well as multidrug resistance in cancers (70), tumor progression, invasion, and metastasis (71). CIC-related tumor dormancy and cellular senescence may also work as an escape mechanism against chemotherapy and other toxic agents. Threatened cells are sheltered from harm within other cells, and dormant cells are also more resistant against harmful agents and treatment drugs (14, 66), meaning that CIC structure-related senescence may be linked to worse prognosis (18).

Tumor cell cannibalism and entosis may promote chromosomal changes that favor tumor progression, such as horizontal DNA transfer and incorporation of protumorigenic traits from the internalized cell to the host cell (40, 47, 52), contributing to a more aggressive cell population (42, 50).

In the same way heterotypical cannibalism favors tumor aggressiveness in several ways, homotypical cannibalism has been related to tumor suppression. In pancreatic adenocarcinoma, the formation of homotypical CIC structures among tumor cells suppressed metastasis (17). This phenomenon was considered serum-dependent, as opposed to the serum-deprivation triggers seen in oncogenic heterotypical cannibalism events (17). Brouwer (38) also reports an increase in cannibalistic activity in serum-rich as opposed to serum-free cultures. Despite not being classified as such at the time, we suggest that this report may have been a case of homotypical cannibalism.

Cell cannibalism has been related to the high rates of cell death in cancer cell populations, being described as a destructive event (38). Despite internalized cells providing nutrients to its cannibal partner, their elimination may lead to suppression of tumor growth (5). Tumor cell cannibalism targeted against cancer cells or mesenchymal cells may result in host-cell destruction (17, 72). Regarding cell senescence and dormancy, the effects of cannibalism on tumor progression may not always be positive. In 3D breast cancer cultures, tumoral cells that cannibalized mesenchymal stem cells became dormant through exosomal transfer of microRNA, resulting in higher expression of the TWIST1 protein and regulation of LOX, JNK and p38. These cells expressed typical inflammatory mediators related to a senescence-associated secretory phenotype (14), which in turn may be responsible for stimulating clearance of these cells through non-professional phagocytosis (73, 74).

#### 2.1.2 Entosis

Entosis has been described as the invasion of a live cell into another which could result in degeneration of the internalized cell (10). It has been reported in benign and malignant cell lines cultured in suspension (10). Morphologically, entosis events resemble typical CIC structures, in which the outer cell has its nucleus displaced towards the periphery in a semilunar shape (75). One of the possible results of entosis is “entotic cell death”, a non-apoptotic cell death pathway (76).

Some authors describe entosis as a subtype or a synonym of cannibalism (50, 77). However, despite their resemblance, entosis involves the active penetration of the internalized cell into its host cell (10, 78), while in cannibalism, the host cell is the one which actively engulfs its “victim”. Secondly, while cannibalized cells undergo apoptosis, in the entosis process, engulfed cell elimination occurs through lysosome-mediated degradation and non-apoptotic cell death (10) and some cells even survive the process (47). Furthermore, entosis is a homotypical CIC event, whereas cannibalism may also involve different types of cells (17, 41, 77). Entosis is sometimes referred to as synonymous to homotypical cannibalism (61). However, while entosis is a response to serum-deprivation, homotypical cannibalism is dependent on serum-factor TGFβ (17). Additionally, homotypical cannibalism is deleterious for cancer cells, while entosis is associated with oncogenesis and tumor progression (11, 17).

The occurrence of entosis is also influenced by the extracellular environment. Overholtzer (10) primarily identified this event *in vitro* in detached mammary epithelial cells. In fact, detachment from the extracellular matrix seems to drive entosis, as a safe mechanism for removal of damaged cells, such as cells unable to trigger apoptosis (61).

Cells in nutrient-deprived environments are more likely to perform entosis. In glucose starvation settings, entosis is induced (68) and a higher frequency of internalized cell destruction has been reported (79). Through entosis, cells can survive in such harsh conditions. Similarly, other harmful events may favor entosis, such as ultraviolet radiation (80) and chemotherapy (6).

The detachment of a cell from the extracellular matrix induces this process by resulting in an unbalanced contractile force in its actin/myosin cytoskeleton, upon loss of integrin signaling, with formation of adherens junctions and Rho GTPase/ROCK signaling activation, which has been previously implicated in aberrant phagocytic activity by non-professional phagocytes (81). Entosis has been described as a ROCK-dependent mechanism of cell engulfment (5) and a mechanism involving the Rho-ROCK-Actin/Myosin pathways is active in the process (11, 50, 77). When cells engulfed through entosis die, they are destroyed by a non-apoptotic lysosomal manner involving autophagy pathway proteins and LC3 expression in the outer vacuole (82). However, upon impairment of lysosome function and autophagy genes, internalized cells may undergo apoptosis or escape the engulfment process (82).

Assembly of the adherens junction associated with an imbalance in myosin II forces boosts the cell into “invading” another with atypical positive E-cadherin and β-catenin staining, as shown by Sun et al. (50, 77), which proposed that activated oncogene Kras may also stimulate entosis in suspended breast cancer cells. On the other hand, inhibition of the mTOR pathway affects the degradation of internalized cells (83), suggesting an association between PI3K/AKT/mTOR activation and the entotic cell profile. Additionally, the ezrin protein is also required for the execution of cell invasion (84).

In the context of entosis, the relationship between the host and the engulfed cell may be defined as one with a “winner” and a “loser”, respectively (50). The “winner” phenotype is characterized by higher mechanical plasticity (50), which is common among cancer cells when compared to their non-neoplastic counterparts - consequently turning them into “loser” or targeted cells for engulfment. In this manner, entosis may be considered a prominent form of cell competition, in which the winner is the cell with higher deformability, which also confers these cells higher metastatic and invasive capacities. The result of this cell selection process is the predominance of a population of cells with oncogene or tumor suppressor mutations, which confer the cells a “winner” profile (58) and favor tumor progression.

The role of entosis in tumor progression and suppression is more complex than cell cannibalism (6). On one hand, it may favor cell survival of starving cells (68) and occur because of chemotherapeutic or other harmful agents (6). Entosis may also promote aneuploidy and accumulation of cell mutations (40). On the other hand, entosis may serve as a clearance mechanism of defective and aberrant cells (77). However, clinical data show a correlation between the number of entosis events and worse clinical prognoses in malignancy (2, 75).

#### 2.1.3 Emperipolesis

Emperipolesis was primarily identified as a transitory and arbitrary passing of a cell through another cell’s cytoplasm, without affecting either of the bodies involved (54). It describes the process of entering, moving within and exiting another cell, having been identified involving healthy - megakaryocytes, monocytes, fibroblasts - and malignant cells (85). Emperipolesis doesn’t necessarily result in the inner-cell’s destruction (31, 47, 86), and may even allow for cell division of the internalized cell while living in its host cell (1, 87). Physiological emperipolesis is found in many contexts, such as leukocyte transcellular migration through the endothelium or neutrophil transportation by macrophages (88).

Like cannibalism and entosis, the definition of emperipolesis varies among different authors. It has been predominantly defined as a heterogeneous cell-cell engulfment of hematopoietic cells (89, 90), while some authors identify emperipolesis as an exclusively heterogenous cell-cell interaction (55). Overholtzer (5), on the other hand, proposed that emperipolesis should be defined as a generic term which describes all cell movements associated with CIC structures, regardless of their formation mechanisms (cannibalism or entosis). Borensztejn et al. (6) classifies emperipolesis as CIC formation through heterotypic cell invasion, usually by a lymphocyte.

In emperipolesis, the inner cell activates the event (54). Phase microscopy of malignant lymphocytes inside macrophages show clear distinction between their membranes and cytoplasm, suggesting that the macrophage reacts to the presence of the inner cell by forming an additional membrane around them (91). The process requires free calcium, adhesion molecules, an actin-based cytoskeleton and high membrane fluidity (87), and is reduced by inhibition of actin polymerization (92).

Emperipolesis may trigger effects on both the inner and the outer cell. For example, the outer cell may be a victim in the process (38, 92, 93), dying *via* lysosome mediated pathway (78). Lymphocytes can increase cytotoxicity by entering tumor cells, which, in some reported cases, are damaged after being invaded by immune cells (87, 94, 95). The internalized cell may survive and escape the host cell, even undergoing mitosis while inside it (1, 87).

However, emperipolesis may result in death of the inner cell. A process that has been described as suicidal emperipolesis (96) is responsible for elimination of autoreactive T cells in the liver (97). In a similar manner, it has been reported that in autoimmune hepatitis, lymphocytes enter hepatocytes and induce their own apoptosis, killing their host cell in the process (97). Finally, some natural-killer cells invade cancerous cells and secrete granzyme B, leading to their own apoptotic cell death (98). This phenomenon was termed emperitosis, which may be considered a form of emperipolesis that results in apoptotic death (98).

### 2.2 Other CIC Reported Cases

Aside from cell cannibalism, entosis and emperipolesis, there are two other relevant processes behind CIC formation: enclysis and phagoptosis. Enclysis results in CIC formation in a manner similar to pinocytosis, but so far it has been considered specific to hepatocytes engulfing T lymphocytes (99). The fate of the inner cell depends on which type of T lymphocyte has been engulfed – regulatory cells being the first to go, through lysosomal digestion (99).

Phagoptosis is considered a form of cell death in which phagocytes engulf viable cells (100). In the case of phagoptosis, the phagocyte is always a macrophage with a phagocytic phenotype, whose engulfing behavior may be enhanced by specific conditions such as inflammatory processes. Differently from cell cannibalism, phagoptosis may occur in pathological as well as physiological conditions, contributing to normal cell turnover. However, like cannibalism, this process ultimately results in lysosomal degradation of the inner cell (6).

CIC structures may also result from well-known physiological processes, which may also be found in the context of cancer, such as phagocytosis and autophagy. However, phagocytosis is recognized by the presence of surface ruffles surrounding the “victim” cell (81), differing from cannibalism (30). Secondly, while macrophages only endocytose dead or dying cells with the intent of cell elimination and antigen-presentation, a cannibal tumor cell targets its living neighbor cells for nutrition (5, 30).

Considering their distinct physiologies, the causes, and consequences of the types of CIC structures in cancer depend on cell types involved and the context of the tumor microenvironment. Several authors have argued that the various engulfment mechanisms are analogous to autophagy regarding cell nutrition, aside from protecting tumor cells from immune surveillance and influencing cancer development (30, 101).

Autophagy has been linked to CIC structures through the transmembrane protein TM9SF4 (102). This protein is activated through mTORC1 suppression in starvation conditions (102). In other words, TM9SF4 enhances phagocytic properties of cells, allowing them to hunt for nutrients – even if by cannibalizing their neighbors. Furthermore, TM9SF4 has been linked to cancer cell metastasis (63, 65). This corroborates to the relation between CIC formation and metastatic cell behavior.

## 3 Cell-in-Cell Events in Oral Squamous Cell Carcinoma

The first report of CIC structures in OSCC was made by Sarode (103). Since then, several reports have followed that not only identified CIC structures but attempted to classify their origin and establish an association with tumor progression. These studies are summarized in **Table 2**.

**Table 2:** Summary of mapped CIC studies in OSCC.

Reference	Main Points
**Sarode etal.** (103)	CIC structures in all degeneration stages were found. First report of complex cannibalism (a cell engulfed within another engulfed cell).
**Jose et al.** (60)	CIC structures found among TNM stages III and IV cases. Positive lymph node cases were found to have more CIC events. Authors suggest as prognostic indicator the relation between size, stage, and frequency of CIC events as well as propensity for metastasis.
**Sarode and Sarode** (47)	Retrospective evaluation of OSCC cases for neutrophil-tumor cell cannibalism. Cases showed adjacent tissue invasion and poor differentiation. CIC events were found in different degeneration stages. Mild to moderate cytoplasmic positivity for CD68 and lysozyme markers was found.
**Jain** (43)	Review on cellular cannibalism focusing on OSCC.
**Sarode, Sarode and Chuodhari** (104)	Analysis of OSCC cases for phagocytic markers CD68 and lysozyme as predictors of cannibalism behavior. OSCC samples with CIC structures were positive for both markers, suggesting their use as cannibalistic markers. Identification of positive staining in cases without CIC structures were considered suggestive of cells with potential cannibal behavior.
**Sarode et al.** (55)	First report of cell emperipolesis in OSCC. Lack of signs of degeneration eliminated the possibility of cell cannibalism. Findings suggest that emperipolesis of NK cells enhance tumor progression.
**Jain et al.** (57)	More CIC events found in metastatic cell lines compared to non-metastatic lines. Authors consider cell cannibalism as a marker of aggressiveness in OSCC.
**Siddiqui et al.** (45)	Cases showed evidence of cannibalism in several stages of degeneration. Authors propose that cancer cell cannibalism is linked to cell de-evolution and retroversion of multicellularity
**Tetikkurt et al.** (105)	Case report of neutrophil emperipolesis by OSCC cells Lack of degeneration evidence eliminated the possibility of cannibalism Authors emphasize emperipolesis as predictor of cancer behavior and therapeutic target.
**Almangush et al.** (51)	Association found between cell-in-cell structures and aggressive histopathological features in early OSCC. Authors suggest cell-in-cell presence as a complementary prognostic indicator.
**Fan et al.** (106)	CIC structures found in 2D co-culture of OSCC cell line and neutrophils. CIC presence negatively associated to prognosis and recurrence-free survival.
**Suwasini et al.** (46)	Cannibalistic cells were identified and linked to the aggressive nature of OSCC.
**Yamazaki et al.** (107)	Case report of OSCC with tumor phagocytosis of neutrophils. CIC structures by formed by neutrophil engulfment supports diagnosis of malignancy in tumor samples

In 2012, Sarode (103) reported the finding of cannibal cells that had cannibalized another cannibal cell – a cell within another within another. This was the first report of such event and was called “complex cannibalism”, suggesting this CIC as an indicative of aggressive behavior in OSCC. The maximum number of complex cannibalism structures were in advanced stage and poorly differentiated cases (103). Two years later, the same group reported the first case of neutrophil-tumor cannibalism in OSCC (47). Neutrophil-rich areas in histological specimens exhibited CIC structures with both partial and complete engulfments, including multiple neutrophils at different degeneration stages. These features are in accordance with the fact that cannibalized cells are destined towards depletion (40, 45, 57) and the previously described complex cannibalism (103).

Histopathological analysis of CIC structures linked to cannibalism showed features of poorly differentiated OSCC and adjacent tissue invasion (47). This evidence further associates CIC findings and a more aggressive and invasive OSCC behavior. More recently, another study reported neutrophil-tumor cannibalism in 2D co-culture *in vitro* studies and retrospective case series analysis (106). Despite adequately identifying CIC findings, the use of 3D culture models would improve the simulation of the tumor microenvironment, allowing for a more realistic mapping of CIC frequency and spatial distribution (108, 109).

OSCC cases were positive for CD68 and lysozyme markers (47). This expression was considered inconclusive but suggested that tumor cells might acquire macrophagic properties and execute lysozyme digestion, which is compatible to previous CIC reports (47, 82). Two years later, the same group performed a retrospective evaluation of genotypic expression of these markers in OSCC samples with and without CIC events (104). The results of 30 analyzed cases were that CIC-associated samples had positive staining for CD68 and lysozyme, suggesting their use as cannibalistic markers. The authors also suggested that some CIC-absent tumor samples with positive cannibalistic markers could be classified as potentially cannibalistic cells, which could become an early diagnosis adjuvant (104). This is further reinforced by association of poorly differentiated OSCC cases with higher expression of CD68 and lysozyme, suggesting their use as a predictor of aggressive cannibalistic behavior (40). A shift in expression of CD4 and CD8 is also described in T-cells involved in CIC events (110).

Several authors emphasize that CIC structures, easily identified in routine H&E staining, could be helpful as prognosis predictors (47, 51, 57, 60, 103, 104, 106). Several studies reported CIC findings and intended to establish an association with the aggressive nature of OSCC. In fact, tumoral CIC structures were frequently linked to aggressiveness in OSCC cases (45–47, 51, 57). The frequency of these events in tumors has been directly related to lymph node involvement, tumor size and stage (47, 60, 103), and in some cases, to tumor grading as well (52), which reinforces its role as a marker of aggressive behavior, prognostic indicator, and predictor of tumor biology in OSCC (1, 10, 46, 51, 57, 60, 103).

Most reports on CIC findings in OSCC were attributed to a cannibalistic origin (46, 47, 57, 103). However, most of these studies did not perform an evaluation of differentially expressed markers that would appropriately classify CIC findings according to their origin (e.g. TM9SF4 in cannibalism cases (63), E-cadherin and LC3 in entosis cases (50, 77, 82). The definition commonly used to identify cannibal structures in OSCC was the same used to describe all CIC structures. Even cells seen in the process of degradation could easily be attributed to cannibalism or entotic origins. Therefore, even though the studies adequately report CIC findings, specific differential identification assays are necessary to correctly define the origin of these structures in OSCC cases.

To our knowledge, there is only one study describing emperipolesis in OSCC, involving lymphocytes within the tumoral cells (55). The absence of inner cell degradation was used as criteria to exclude the possibility of cell cannibalism. In regard to entosis, the broad and unreliable use of the terms related to CIC formation make it hard to rule out its occurrence. Cell detachment from the extracellular matrix and loss of cellular adhesion, which promote entosis, recall another relevant process common in cancer: the epithelial-mesenchymal transition (111). These events have been reported frequently in OSCC, and participation of markers common to both processes, such as E-cadherin, have been observed in this type of cancer (112). Further research is important to correctly identify the mechanism of entosis as a part of OSCC biological behavior (e.g. cell plasticity, invasion, and clearance).

## 4 Conclusion

Cases of CIC structures can no longer go unnoticed in cancer, as was the case for several years. It is already understood that these phenomena profoundly affect tumor development due to their pro-tumorigenic effects and their suppressive effects. In the context of the OSCC, an aggressive cancer with few therapeutic options, understanding the biology of CIC structures is essential for its use in early diagnosis and incorporation as a possible therapeutic target. Further studies in this area may allow the isolation of these pro-tumor and anti-tumor effects, transforming these CIC events into tools for combating the tumor, either through their induction or suppression. Even so, it is essential to consolidate up-to-date scientific methods for simulating and analyzing these events, such as three-dimensional culture models and tools for gene editing and cell therapy, for an adequate management of CIC structures.

## Author Contributions

Conceptualization: LS, BS, DL, CG. Funding acquisition: DL, CG. Project administration: DL, CG. Writing—original draft: LS, DL, CG. Writing—review and editing: LS, DL, CG. All authors contributed to the article and approved the submitted version.

## Funding

We acknowledge funding from the Academy of Medical Sciences/Newton Advanced Fellowship Grant (NAFR12\1035) (to CG and DL) and Brazilian research financial institution the National Council for Scientific and Technological Development (CNPq) (308276/2019-1) (to CG).

## Conflict of Interest

The authors declare that the research was conducted in the absence of any commercial or financial relationships that could be construed as a potential conflict of interest.

## Publisher’s Note

All claims expressed in this article are solely those of the authors and do not necessarily represent those of their affiliated organizations, or those of the publisher, the editors and the reviewers. Any product that may be evaluated in this article, or claim that may be made by its manufacturer, is not guaranteed or endorsed by the publisher.
